# The Book World of Medicine and Science

**Published:** 1896-10-31

**Authors:** 


### The Book World of Medicine and Science.

The Causes and Treatment of Rheumatoid Arthritis. By Samuel Hyde, M.D. (London: John Beal and Son. 1896. 8vo., 80 pages, 3s. 6d.)

In this little manual of 80 pages the etiology, pathology, symptomatology, and treatment of the condition are all dealt with—the treatment coming in for the lion's share of the author's attention. A satisfactory definition of the disease has always been a matter of difficulty, but that supplied by Dr. Hyde appears to meet all the requirements of the case; it runs as follows: "Rheumatoid arthritis is a tropho-neurosis of joints, unassociated with any systemic disorder, and probably dependent upon some central nervous lesion." It seems almost a pity that Dr. Hyde could not accept this definition as a working basis for the rest of the points, pathological, therapeutic, and otherwise, with which he deals. He allows himself, however, to be blown about by side winds, and is inclined to hedge and cover his statemonts against the discovery of some even more satisfactory explanation of the condition; for instance, on page 33, he remarks, "Personally I am inclined to the view that rheumatoid arthritis, and many chronic joint affections, are due to perverted internal secretion, partly of a toxic character," &c. Again he pictures the pathological history by a typical case, page 35: "We should have first nervous depression, then would follow perverted internal secretion, and auto-intoxication of the blood and circulating fluids of the body," &c. Why, if he believes this, should he add in the next page, "My own clinical experience has long suggested to my mind such an organism (micro-organism described by Bannatyne, Wohlmann, and others) in rheumatoid arthritis"? This uncertainty on fundamental points does not inspire confidence in the readers. But there are many useful points to be learned by reading these pages. As far as treatment goes, Dr. Hyde appears to us to be quite sound in his views —he recommends good feeding, warmth, bracing climate, baths of blood temperature, massage, and arsenic. He also inclines to the use of animal juices, it having occurred to him that the administration of a suitable extract prepared from the articular cartilages and synovial membranes of healthy animals might be useful in restoring the physiological equilibrum. At the time of writing, Dr. Hyde assures us that he has used these extracts for some months past, and in many cases there has been a distinct improvement in the joint affections. More remarkable things have happened than that his views on this point should prove correct; but at present we should like to see the method independently tested before giving adherence to it.

Food in Health and Disease. By J. Burney-Yeo, M.D., F.R.C.P. New and Revised Edition. (London: Cassell and Co., 1896. Price 10s. 6d.)

In the treatment of disease there are many pitfalls, and towards none of them is the way more seductive or the path more easy than towards that most common error of placing one's reliance upon physic and neglecting the circumstances and surroundings of the patient. Among the circumstances which have the most marked influence both in the production of disease and in its cure diet stands pre-eminent, and Dr. Burney Yeo has done well to put together in one volume a general review of the whole subject of dietetics. The choice of food, its preparation, its digestion, and its assimilation are matters which demand the most careful consideration at the hand of the medical man, and it is the fact that the choice and preparation of food stuffs are so often left to cooks and housekeepers, while the doctor is asked merely to rectify by medicine any evils which may arise from its unsuitability, that makes many diseases of nutrition so difficult to treat. Dr. Yeo, then, recognising their interdependence, treats them as but different parts of one subject. He describes the various substances used as food and drink, gives their several food values, deals with the scientific basis of dietaries and rations, giving examples from many sources, and tells us about the kinds of diet required by different ages and conditions of life and about the various methods of cooking in common use.

Turning then to the second part of the book, which is devoted to "Food in Disease," we find a careful description of the diet which has been found most suitable in various diseases, interspersed with much that is interesting and with many "tips," if we may so call them, as to the management of the maladies in question. The range of the work is so extensive that it would only be misleading to give quotations from any special part; sufficient to insist, as we do again, that the subject of food, both in health and disease, is one of very great importance, and to say that in this work Dr. Burney Yeo has dealt with it in a most able and instructive manner.

The Objects and Limits of Operations for Cancer, Being the Lettsomian Lectures for 1896. By W. Watson Cheyne, M.B., F.R.S., F.R.C.S. (London: Baillière, Tindall, and Cox. 1896. Price 5s.)

We noticed these admirable lectures at the time of their delivery. There is no doubt that the more hopeful view which they have held out in regard to permanent cure in certain forms of cancer has already had a stimulating influence on many surgeons, giving them confidence in undertaking extensive operations wherever there is a chance that by such means the whole of the disease can be removed. The key to Mr. Watson Cheyne's recommendations is thoroughness, and his results go far to prove the correctness of his teaching. At the same time, it must be recognised that this very thoroughness renders operative work for the relief of cancer a much more serious affair than it has often been considered. The large incisions, the deep extension of the wound so as to remove the whole of the infected layers, and the extirpation along with these, not merely of infected glands, but of masses of such glands as are known to be likely to be involved, renders even such an operation as the removal of a cancer of the breast a matter of no small gravity, and one not to be undertaken by a surgeon unless he is so confident in his methods as to feel sure of being able to ensure asepsis. The thesis maintained by Mr. Watson Cheyne in these lectures may be stated thus: The primary object of operation in cancer is the prolongation of the patient's life and the alleviation of his local trouble, and these results are in most cases best attained by aiming, wherever it is possible, at the *cure* of the disease, notwithstanding the dangers of the larger operations involved. With this most of us will agree; in fact, many in agreeing will also maintain that Mr. Watson Cheyne, in urging free removal, is but an exponent of a drift in professional opinion which has been showing itsel in the practice of many men for a considerable time. Whil recognising, however, that this feeling exists, we must offer to Mr. Watson Cheyne the very high praise which is his due for the admirable results which have followed his efforts to remove the disease as completely as was possible.

